# Predicting 365-Day Lower-Limb Amputation at Discharge After Diabetes-Related Foot Ulcer Hospitalization: Development and Temporal Validation

**DOI:** 10.1093/ofid/ofag673

**Published:** 2026-10-09

**Authors:** Yuriko Fukuta, Mayar Al Mohajer

**Affiliations:** Department of Medicine, Section of Infectious Diseases, Baylor College of Medicine, One Baylor Plaza, Houston, Texas, USA; Department of Medicine, Section of Infectious Diseases, Baylor College of Medicine, One Baylor Plaza, Houston, Texas, USA; Section of Infectious Diseases, Baylor St. Luke's Medical Center, Houston, Texas, USA; Nuffield Department of Primary Care Health Sciences, University of Oxford, Oxford, UK

**Keywords:** competing risks, diabetes-related foot ulcer, lower-limb amputation, prognostic model, temporal validation

## Abstract

**Background:**

Patients discharged after diabetes-related foot ulcer hospitalization remain at risk of limb loss. We developed and temporally validated discharge-time models for 365-day amputation, comparing clinical-plus-discharge predictors with a fuller set including contextual information.

**Methods:**

Epic Cosmos provided 431 493 development patients discharged in 2016–2024 and 36 823 temporal-validation patients discharged in January–June 2025. Cause-specific Cox models estimated any and major amputation with death as a competing event. Model C included clinical, contextual, and discharge variables; Model D omitted contextual variables. Models were compared using discrimination, calibration, and decision curves.

**Results:**

Observed 365-day cumulative incidences were 19.3% for any amputation and 6.4% for major amputation. Model C areas under the receiver operating characteristic curve were 0.710 and 0.737, compared with 0.711 and 0.736 for Model D. Corresponding calibration slopes were 1.003 and 1.026 for Model C and 1.010 and 1.025 for Model D. Brier scores were similar. Gangrene and peripheral artery disease had the largest joint Wald statistics in Model C. Its highest-risk quintile contained 40.6% of any-amputation and 49.6% of major-amputation events.

**Conclusions:**

Models C and D showed similar performance, with moderate discrimination and risk enrichment. These findings do not establish superiority of the fuller model, irrelevance of contextual factors, or improved patient outcomes. Independent validation, local recalibration, and prospective impact evaluation are required before clinical use.

## Teaser

Clinical-plus-discharge and fuller contextual models showed similar performance for 365-day amputation after diabetes-related foot ulcer hospitalization. Risk enrichment does not establish clinical benefit; independent validation and prospective impact evaluation are required.

Diabetes-related foot ulceration is frequently complicated by infection, osteomyelitis, ischemia, hospitalization, amputation, and premature death [1, 2]. Among patients discharged without index amputation, neuropathy, peripheral artery disease, impaired healing, and residual or recurrent infection may sustain risk [2–4]. Social and health-system conditions also matter: rural residence and racial inequities have been associated with major amputation or death among patients hospitalized with foot ulcers [5].

International Working Group on the Diabetic Foot and Infectious Diseases Society of America guidance emphasizes assessment of infection and ischemia, source control, revascularization when indicated, offloading, wound care, and coordinated follow-up [3, 4]. We use intensified follow-up to mean earlier or more coordinated vascular, wound, podiatric, infectious diseases, and home-health assessment according to clinical need—not a tested visit schedule or an infectious diseases-only intervention.

Existing prediction models differ in populations, predictors, outcome definitions, and performance, and many have limited validation or high risk of bias [6]. The question here is whether contextual information improves prediction beyond routine clinical and discharge information at a 365-day horizon, not whether social determinants are associated with amputation. We developed and temporally validated models in Epic Cosmos [7], comparing author-selected predictor sets and assessing discrimination, calibration, risk enrichment, and decision-analytic net benefit.

## METHODS

### Study Design and Data Source

This retrospective prognostic study used deidentified electronic health record data from participating US Epic Cosmos organizations [7], not a census of US hospitals. A source organization is a contributing data unit and may represent multiple facilities. Each patient contributed one eligible inpatient hospitalization. The original extraction was completed on 28 July 2026, and the administrative outcome-data cutoff was 30 June 2026. The development and temporal-validation cohorts contained 262 and 250 source-validation groups, respectively; the 2015 sensitivity cohort contained 130. These are analytic grouping counts, not counts of individual hospitals or additive counts of unique organizations (Supplementary Table 14).

### Study Population and Source Coverage

Adults aged 18 years or older contributed their first qualifying US inpatient hospitalization with discharge from 1 January 2015 through 30 June 2025. Index deaths were excluded first, followed by index amputations among survivors. Eligibility required the entire index stay to fall within the applicable source-complete transfer interval: admission on or after its start and discharge on or before its end, with both boundaries present. For multisource encounters, the latest start and earliest end across contributing sources defined the interval. A prior-year encounter was not mandatory. This source-period rule does not establish continuous enrollment, complete care retention, or uniform coding (Supplementary Table 15).

Discharges in 2016–2024 formed the development cohort; 1 January–30 June 2025 formed temporal validation; and 2015 was reserved for sensitivity analysis. The available eligible population determined sample size. All model results reported here retain the original analysis cohort; no model was refitted for this manuscript revision.

### Outcomes and Follow-up

The primary outcome was first subsequent lower-limb amputation during days 1–365 after discharge; the secondary outcome was first major lower-limb amputation. The implemented definition used Current Procedural Terminology lists and ICD procedure-code rules, including an ICD-10-PCS prefix/body-part algorithm; description-only and anesthesia matches were removed before event assembly (Supplementary Table 15). Death recorded before the corresponding endpoint was a competing event. Observation ended at the outcome, recorded death, last observable date, source-complete transfer end, day 365, or 30 June 2026, whichever occurred first. Potential administrative follow-up therefore did not guarantee complete individual follow-up. Community and outside-system deaths could be missed.

### Candidate Predictors

We compared author-defined predictor sets. Model A included age, sex, routine laboratory values, body mass index, length of stay, osteomyelitis, cellulitis or soft-tissue infection, necrotizing fasciitis, sepsis, gangrene, peripheral artery disease, neuropathy, end-stage kidney disease or dialysis, prior amputation, and prior revascularization. Model B added preferred language, insurance, primary-care-provider status, Social Vulnerability Index (SVI), Rural-Urban Commuting Area (RUCA), and US region [8, 9]. Model C added deep debridement, index revascularization, discharge disposition, home-health referral, and hospice. Model D combined clinical and discharge variables without contextual variables. Model E added race and ethnicity to Model C for sensitivity analysis. Prior ulcer history was intentionally omitted from the extraction specification. Smoking was considered during planning but excluded from the primary predictor sets because of concerns about sparse flowsheet documentation and variation in institutional recording. Neither omission establishes a lack of prognostic value (Supplementary Table 1).

### Data Quality and Preprocessing

Age was calculated from birth date at index discharge, with age ≥ 18 required by SQL eligibility. Before modeling, continuous values outside specified plausibility limits were set to missing. The original audit identified 5 ages above 110 years—4 in development and 1 in temporal validation; no age was missing before cleaning. Records were retained using the same median-imputation and missingness-indicator approach applied to other continuous predictors, rather than an additional age-based complete-case exclusion. Continuous predictors used 3-degree-of-freedom natural cubic splines when feasible; categorical predictors retained explicit unknown levels. Development-fitted preprocessing was frozen for temporal validation. Supplementary Table 16 reports continuous missingness, categorical unknown or combined other/unknown levels, and completeness of the derived binary representation; a coded zero does not establish clinical assessment. No univariable screening or automated stepwise selection was performed (Supplementary Tables 2–4 and 16).

### Model Development and Absolute-Risk Estimation

For each outcome and predictor set, separate cause-specific Cox models for amputation and death were combined to estimate 365-day cumulative incidence [10]. Predictions used the uncentered design vector; the all-zero reference vector is not the average patient. Supplementary Data SD1–SD3 provide released Model C coefficients, design columns, and cumulative baseline hazards; field definitions are in Supplementary Tables 5 and 6. The nonestimable age_missing and language_group__Unknown columns were fixed at coefficient zero in the final Model C specification. This does not establish that these predictors had no missing observations.

Following development and temporal validation, Models C and D were refitted in the combined 2016–June 2025 cohort for final parameter estimation. Every reported performance estimate instead uses development-fitted models without temporal-validation refitting. Model C remains the full model shown in detailed predictor and subgroup displays, not a clinically preferred model. Only its complete coefficient set is included in Supplementary Data SD1; Model D cannot be implemented from the shared design and baseline-hazard files alone. Availability of one coefficient set is a reporting distinction, not evidence of superior prediction. The final-refit preprocessing recipe is supplied in Supplementary Tables 17 and Data SD9, separately from the development-fitted recipes used to calculate validation performance. Repeated internal knot values for HbA1c, white blood cell count, and neutrophil percentage were retained in the fitted spline specification rather than deduplicated.

### Validation and Model Performance

Frozen models were applied to temporal validation. Models C and D also underwent 5-fold source-grouped cross-validation, with preprocessing repeated within each training fold (Supplementary Figure 3, Tables 7 and 8, and Data SD4 and SD5). At 365 days, we estimated inverse-probability-of-censoring weighted area under the receiver operating characteristic curve (AUC), area under the precision-recall curve, and Brier score [11, 12]; calibration intercept, slope, and observed-to-expected (O/E) ratio; and observed Aalen–Johansen cumulative incidence by risk decile. Ninety-five percent confidence intervals used 500 source-cluster bootstrap samples. Paired comparisons used the same resamples (Supplementary Table 9).

### Decision Analysis, Subgroups, and Sensitivity Analyses

Decision curves evaluated net benefit under the selected threshold probabilities [13]. The highest-risk 10% and 20% were summarized by sensitivity, Aalen–Johansen positive predictive value, and patients evaluated per event identified (Supplementary Table 10). These are retrospective operating characteristics, not estimates of treatment effects. Model C subgroup reporting required at least 200 patients, 20 events, 20 nonevents, and 5 source-validation groups (Supplementary Table 11 and Data SD6). Infection analyses assessed osteomyelitis and any coded infection (Supplementary Table 12). Sensitivity analyses excluded hospice, added 2015 to development, and added race and ethnicity (Supplementary Tables 13 and 14).

### Software, Ethics, and Patient Involvement

Analyses used R, including survival and splines. Direct identifiers were unavailable and patient-level predictions remained within the approved Cosmos environment. Under the institutional policy reported for this study, analysis of fully deidentified data was not considered human subjects research; institutional review board review and informed consent were not required. Patients and the public were not involved. Reporting was informed by TRIPOD + AI, with PROBAST + AI informing risk-of-bias and applicability considerations [14, 15]. ChatGPT (OpenAI) assisted with code review, language editing, and formatting; it was not used to generate patient observations or replace missing empirical results.

## RESULTS

### Cohort Assembly and Characteristics

In the analysis dataset, 701 244 first qualifying discharges yielded 506 629 eligible patients after exclusion of 20 030 index deaths and 174 585 index amputations among survivors. Source coverage excluded 9702 (1.92%), leaving 496 927 patients: 28 611 in the 2015 sensitivity cohort and 468 316 in the primary cohort, comprising 431 493 development and 36 823 temporal-validation patients (Figure 1). Median age was 65 years in development and 68 years in temporal validation. Temporal-validation patients more often had a non-English preferred language, osteomyelitis, cellulitis or soft-tissue infection, sepsis, gangrene, peripheral artery disease, and index revascularization (Table 1; Supplementary Table 3).

**Figure 1.**
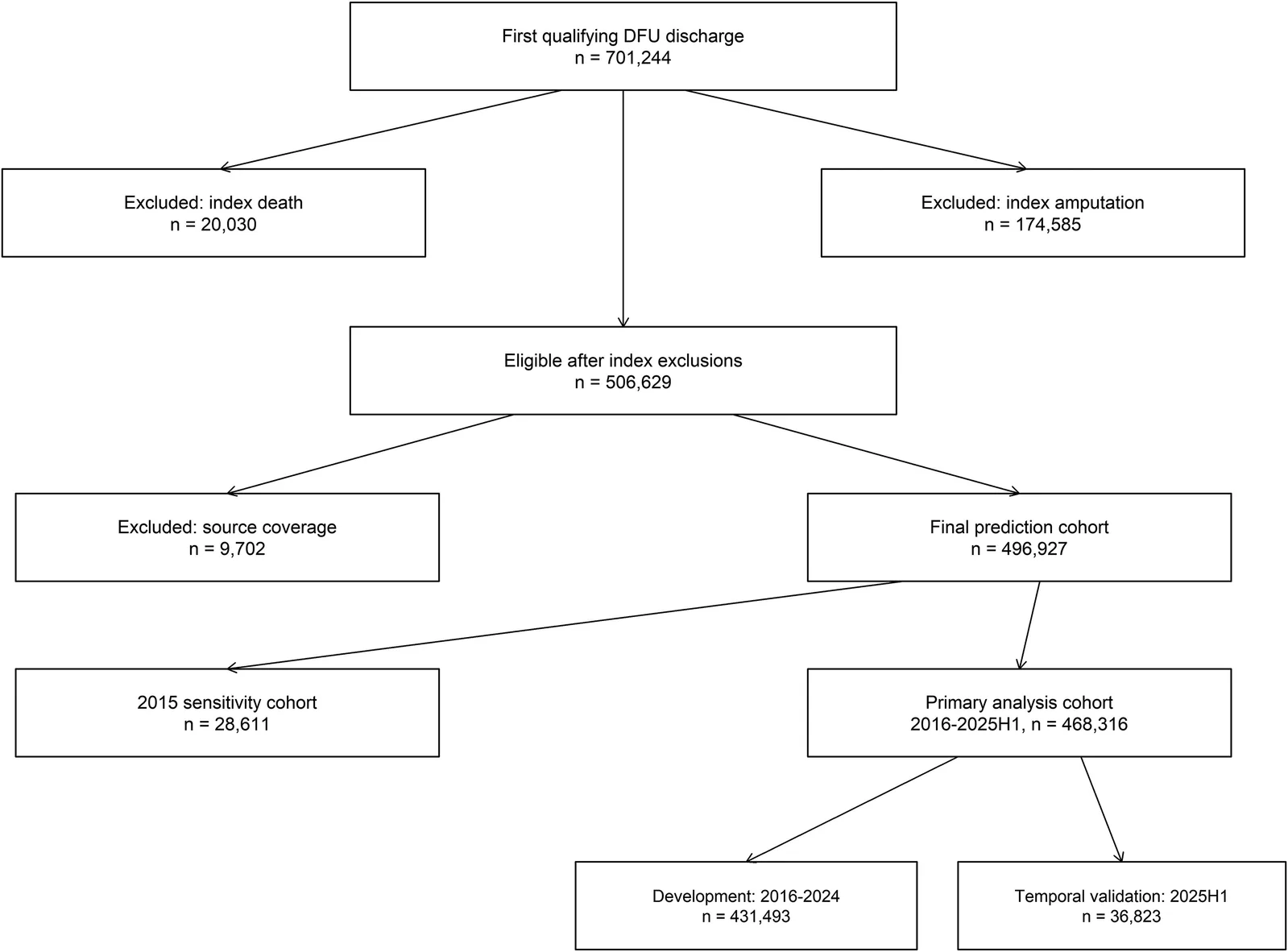
Cohort flow in the locked model extraction. Index death and index amputation among survivors were applied as mutually exclusive exclusions. After source coverage, the cohort was divided into the 2015 sensitivity cohort and the primary cohort: development in 2016–2024 and temporal validation in January–June 2025. DFU, diabetes-related foot ulcer; 2025H1, January 1–30 June 2025.

**Table 1.** Selected Characteristics of the Development and Temporal-Validation Cohorts

Characteristic	Development, 2016–2024 (n = 431 493)	Temporal Validation, January–June 2025 (n = 36 823)
Patients	431 493	36 823
Age, years	65.0 (56.0–75.0)	68.0 (58.0–77.0)
Body mass index, kg/m^2^	30.4 (25.6–36.4)	29.4 (25.0–35.1)
Index length of stay, days	5.0 (3.0–9.0)	6.0 (3.0–9.0)
Social Vulnerability Index	0.71 (0.43–0.89)	0.71 (0.44–0.89)
Sex: female	148 785 (34.5)	12 328 (33.5)
Sex: male	282 689 (65.5)	24 492 (66.5)
Sex: other or unknown	19 (0.0)	3 (0.0)
Preferred language: non-English	52 011 (12.1)	6394 (17.4)
Insurance: Medicaid	61 351 (14.2)	4834 (13.1)
Insurance: Medicare	247 285 (57.3)	21 587 (58.6)
Insurance: self-pay/uninsured	8196 (1.9)	673 (1.8)
SVI band: Q4 (0.75–1.00)	188 646 (43.7)	16 485 (44.8)
SVI missing	7917 (1.8)	528 (1.4)
RUCA: rural	13 377 (3.1)	1071 (2.9)
Osteomyelitis	124 666 (28.9)	11 545 (31.4)
Cellulitis/soft-tissue infection	226 826 (52.6)	20 231 (54.9)
Necrotizing fasciitis	4163 (1.0)	394 (1.1)
Sepsis	98 812 (22.9)	8933 (24.3)
Gangrene	67 585 (15.7)	6543 (17.8)
Peripheral artery disease	173 619 (40.2)	15 893 (43.2)
End-stage kidney disease/dialysis	49 175 (11.4)	3775 (10.3)
Prior amputation	67 029 (15.5)	5135 (13.9)
Prior revascularization	28 335 (6.6)	2362 (6.4)
Index-admission revascularization	32 285 (7.5)	3342 (9.1)
Discharge disposition: home	275 832 (63.9)	23 187 (63.0)
Discharge disposition: skilled nursing facility	83 940 (19.5)	7241 (19.7)
Discharge disposition: rehabilitation	12 339 (2.9)	1082 (2.9)
Discharge disposition: hospice	9832 (2.3)	912 (2.5)

Values are median (interquartile range) or n (percent). Table 1 presents selected characteristics; expanded distributions are in Supplementary Table 3 and missingness/encoded completeness in Supplementary Table 16. SVI bands use fixed national percentile cut points.

Abbreviations: RUCA, Rural-Urban Commuting Area; SVI, Social Vulnerability Index.

### Model Development and Internal Validation

In Model C, gangrene and peripheral artery disease had the largest joint Wald χ² statistics for both outcomes. Length of stay, index revascularization, osteomyelitis, and disposition ranked prominently for any amputation; prior revascularization, prior amputation, and kidney failure ranked prominently for major amputation (Supplementary Figures 1 and 2). These statistics summarize association with the modeled cause-specific hazard, not causal effects or the change in AUC after removing a predictor. Source-grouped out-of-fold AUCs were 0.699 and 0.731 for Model C and 0.699 and 0.729 for Model D for any and major amputation, respectively (Supplementary Data SD4).

### Temporal-Validation Performance

Among all 36 823 temporal-validation patients, 6339 had any amputation and a separate 5195 died before any amputation. For the major-amputation endpoint, 2094 had major amputation and 5588 died before major amputation. The competing-death counts are not subsets of patients with the corresponding amputation. Observed 365-day cumulative incidences were 19.3% (95% CI, 18.7%–19.9%) and 6.4% (6.1%–6.8%), respectively; these censoring-adjusted estimates differ from crude event proportions.

For any amputation, AUC was 0.710 (95% CI, 0.701–0.718) for Model C and 0.711 (0.702–0.720) for Model D. Calibration slopes were 1.003 (0.958–1.054) and 1.010 (0.963–1.059), and Brier scores were 0.142 for both. For major amputation, AUCs were 0.737 (0.726–0.749) and 0.736 (0.724–0.749), slopes 1.026 (0.972–1.080) and 1.025 (0.970–1.078), and Brier scores 0.057 for both. O/E ratios were 1.070 and 1.072 for any amputation and 0.949 and 0.955 for major amputation, for Models C and D, respectively (Table 2; Figure 2).

**Figure 2.**
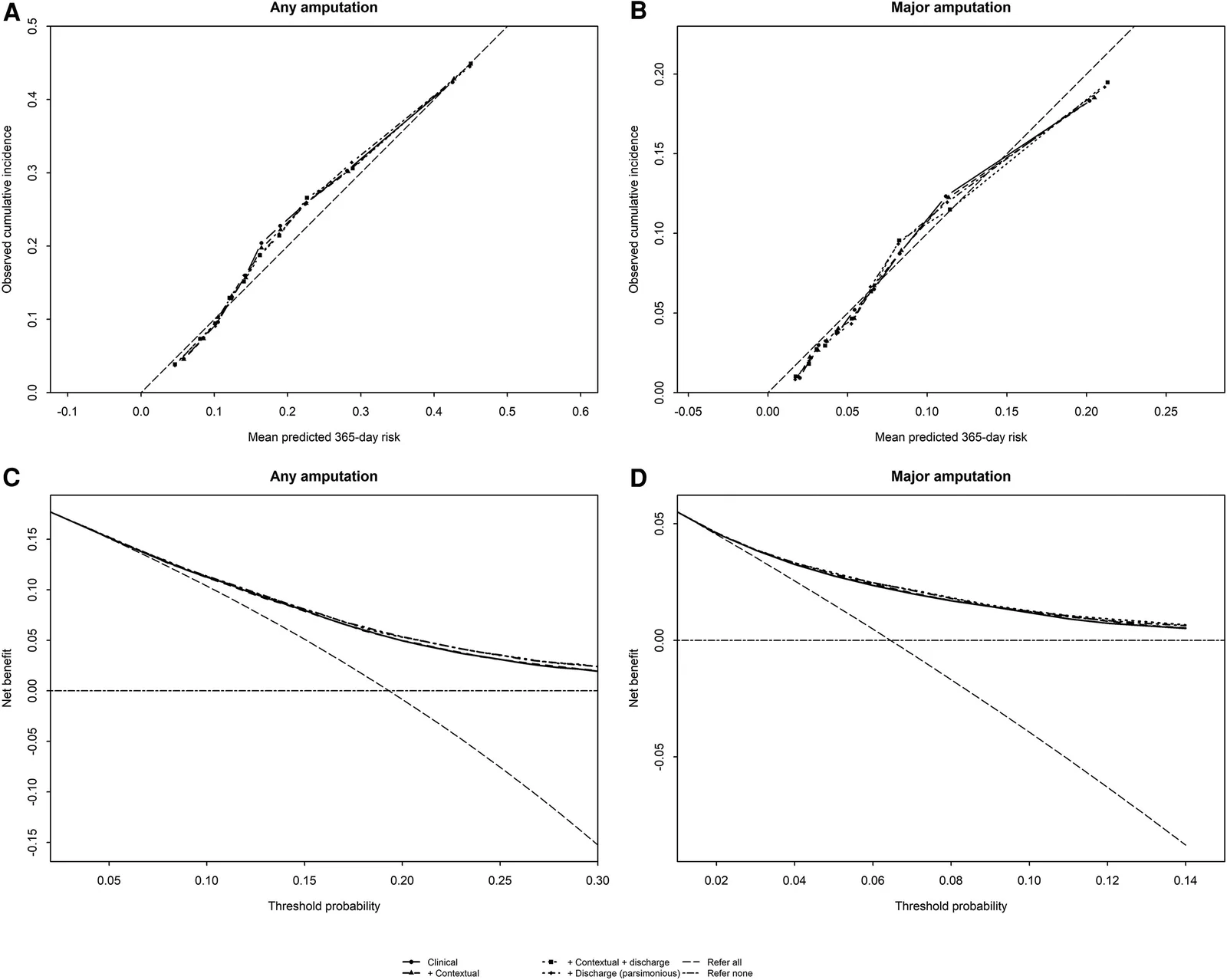
Temporal-validation calibration and decision curves. Panels *A* and *B* show observed Aalen–Johansen cumulative incidence by decile of predicted 365-day risk for any and major amputation, respectively. Panels *C* and *D* show net benefit across the evaluated decision thresholds, with refer-all and refer-none comparators. Models A, B, C, and D denote clinical; clinical plus contextual; clinical plus contextual plus discharge; and clinical plus discharge predictors, respectively. Decision curves do not establish improved patient outcomes.

**Table 2.** Temporal-Validation Performance of the Author-defined Models

Measure	Model A	Model B	Model C	Model D
A. Any amputation				
Patients, n	36 823	36 823	36 823	36 823
Events, n	6339	6339	6339	6339
Competing deaths, n	5195	5195	5195	5195
Observed risk, %	19.3 (18.7–19.9)	19.3 (18.7–19.9)	19.3 (18.7–19.9)	19.3 (18.7–19.9)
Predicted risk, %	18.0 (17.8–18.3)	18.0 (17.8–18.3)	18.1 (17.8–18.3)	18.0 (17.8–18.3)
Observed/expected	1.074 (1.043–1.109)	1.071 (1.040–1.106)	1.070 (1.040–1.105)	1.072 (1.041–1.108)
AUC	0.700 (0.691–0.709)	0.699 (0.690–0.708)	0.710 (0.701–0.718)	0.711 (0.702–0.720)
AUPRC	0.349 (0.336–0.363)	0.349 (0.336–0.363)	0.365 (0.352–0.381)	0.366 (0.353–0.381)
Brier score	0.144 (0.140–0.148)	0.144 (0.140–0.148)	0.142 (0.139–0.146)	0.142 (0.139–0.146)
Calibration intercept	0.088 (0.050–0.133)	0.079 (0.043–0.124)	0.071 (0.033–0.119)	0.078 (0.039–0.125)
Calibration slope	1.020 (0.970–1.071)	1.013 (0.963–1.066)	1.003 (0.958–1.054)	1.010 (0.963–1.059)
B. Major amputation				
Patients, n	36 823	36 823	36 823	36 823
Events, n	2094	2094	2094	2094
Competing deaths, n	5588	5588	5588	5588
Observed risk, %	6.4 (6.1–6.8)	6.4 (6.1–6.8)	6.4 (6.1–6.8)	6.4 (6.1–6.8)
Predicted risk, %	6.8 (6.6–6.9)	6.8 (6.6–7.0)	6.8 (6.6–7.0)	6.8 (6.6–6.9)
Observed/expected	0.954 (0.903–1.008)	0.948 (0.898–1.002)	0.949 (0.897–1.004)	0.955 (0.904–1.011)
AUC	0.726 (0.713–0.738)	0.729 (0.716–0.741)	0.737 (0.726–0.749)	0.736 (0.724–0.749)
AUPRC	0.159 (0.147–0.176)	0.162 (0.149–0.177)	0.168 (0.155–0.184)	0.170 (0.157–0.188)
Brier score	0.058 (0.055–0.061)	0.058 (0.055–0.061)	0.057 (0.055–0.060)	0.057 (0.055–0.061)
Calibration intercept	−0.060 (−0.121 to 0.004)	−0.067 (−0.130 to −0.007)	−0.071 (−0.134 to −0.006)	−0.063 (−0.127 to 0.004)
Calibration slope	1.022 (0.965–1.075)	1.025 (0.966–1.080)	1.026 (0.972–1.080)	1.025 (0.970–1.078)

Parentheses contain 95% confidence intervals from 500 source-cluster bootstrap replicates, with development-fitted models held fixed. Observed risk is Aalen–Johansen cumulative incidence, not the crude event proportion. AUC, AUPRC, and Brier score use inverse-probability-of-censoring weighting at 365 days. Ideal calibration intercept and slope are 0 and 1. A: clinical; B: clinical plus contextual; C: clinical plus contextual plus discharge (full model); D: clinical plus discharge (parsimonious comparator). Smaller Brier scores indicate better overall accuracy.

Abbreviations: AUC, area under the receiver operating characteristic curve; AUPRC, area under the precision-recall curve.

Model C-minus-D AUC differences were −0.00140 (95% CI, −0.00243 to −0.00028) for any amputation and 0.00098 (−0.00138–0.00420) for major amputation. Paired precision-recall and Brier score difference intervals included zero (Supplementary Table 9). Models A and B had any-amputation AUCs of 0.700 and 0.699 and major-amputation AUCs of 0.726 and 0.729 (Table 2).

### Risk Enrichment and Decision Curves

The highest-risk 10% under Model C contained 24.3% of any-amputation and 31.4% of major-amputation events, with censoring-adjusted positive predictive values of 44.9% and 19.5%. The highest-risk 20%—one quintile, not a quartile—contained 40.6% and 49.6%, with positive predictive values of 37.8% and 15.5% (Supplementary Table 10). Model curves showed positive net benefit across the displayed thresholds, with small differences between Models C and D (Figure 2). Model C quintile cumulative-incidence curves remained ordered through day 365 (Figure 3).

**Figure 3.**
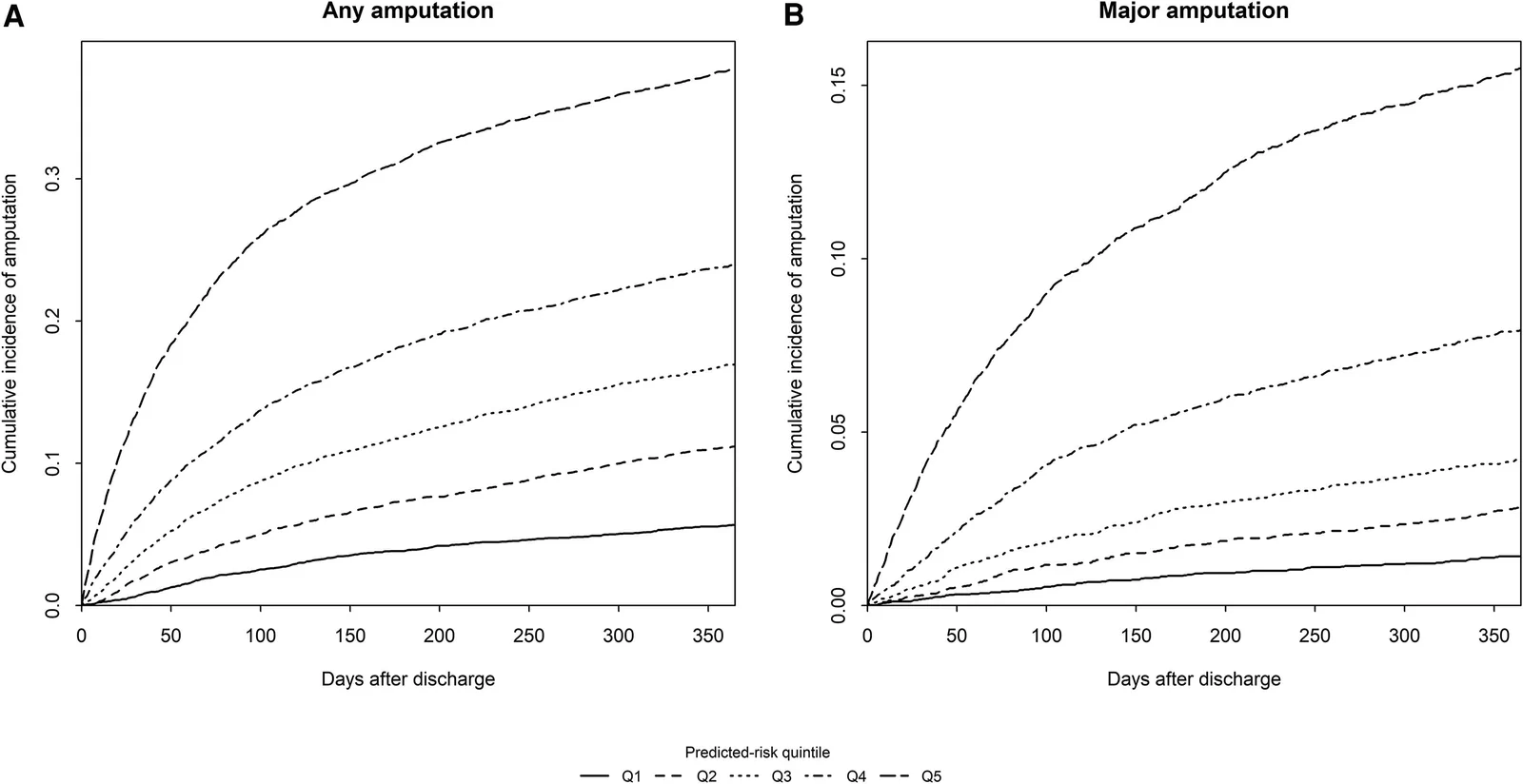
Observed cumulative incidence by Model C predicted-risk quintile in temporal validation. Panels *A* and *B* show Aalen–Johansen cumulative incidence of any and major amputation, respectively. Q1 and Q5 are the lowest- and highest-risk quintiles. Model C includes clinical, contextual, and discharge variables; its display here does not imply superiority over Model D.

### Subgroup and Sensitivity Analyses

Across reportable subgroups, Model C AUCs ranged from 0.604 to 0.732 for any amputation and 0.583 to 0.801 for major amputation, compared with 0.710 and 0.737 overall; the 6 lowest any-amputation estimates were for self-pay/uninsured (0.604), American Indian/Alaska Native (0.620), unknown-race (0.645), unknown-ethnicity (0.654), commercial-insurance (0.665), and non-English-language (0.673) groups, with estimates of 0.681 for unknown RUCA and 0.685 for other/unknown insurance. Relative to overall O/E ratios of 1.07 for any and 0.95 for major amputation, major-amputation O/E was 1.12 in Black and 0.86 in White patients, other-government insurance had O/E ratios of 1.35 and 1.45 for any and major amputation, respectively, and other/unknown insurance had an any-amputation O/E of 1.28; these are descriptive contrasts, with complete subgroup estimates, reportability flags, and confidence intervals in Supplementary Data SD6 (Supplementary Figures 4–7).

Among 27 644 patients with any coded infection, Model C AUCs were 0.697 for any and 0.734 for major amputation; among 11 545 with osteomyelitis, they were 0.656 and 0.696. Corresponding any-amputation O/E ratios were 1.102 and 1.091 (Supplementary Table 12). Sensitivity analysis AUCs across Models C, D, and E ranged from 0.705 to 0.711 for any and 0.734 to 0.741 for major amputation (Supplementary Table 13).

## DISCUSSION

Clinical-plus-discharge Model D and fuller contextual Model C showed similar predictive performance in this large cohort. Both had moderate discrimination, calibration slopes near one, and small average calibration departures. The statistically nonzero any-amputation AUC difference slightly favored Model D, but its magnitude does not establish a clinically important advantage. These comparisons do not establish formal equivalence.

These findings complement rather than replace detailed bedside assessment. IWGDF guidance supports classification for clinical characterization rather than endorsing an existing system to predict an individual ulcer outcome [16]. Earlier infected-ulcer and wound-clinic studies used different populations and measurements [17–19]. Differences in setting, endpoints, follow-up, and competing-risk handling preclude direct claims that this model outperforms or underperforms earlier tools. Its contribution is a scalable discharge-time comparison with temporal and source-grouped validation, not demonstration of a new high-risk mechanism or improved outcomes.

The available contextual variables did not consistently improve aggregate performance. This does not justify removing social determinants from care or other prediction models. Area-level measures, recorded language and insurance, missingness, and limited functional forms may inadequately represent individual barriers or interactions. Context may influence access to treatment and performance in particular settings without substantially changing overall AUC [5]. Model C remains a full specification for comparison, whereas Model D is a simpler candidate for further evaluation; neither is endorsed for deployment. Better contextual measurement could be tested prospectively without assuming that it will improve prediction.

The top-quintile enrichment suggests a population in which a multidisciplinary intervention could be studied, not a validated referral rule. Decision-analytic net benefit depends on the threshold and assumed consequences of decisions; it does not demonstrate prevention of amputations, acceptable workload, cost-effectiveness, or benefit over clinician judgment. Prospective comparative evaluation must establish whether model-guided follow-up changes care and outcomes before either model is used for triage.

Important limitations arise from EHR-based ascertainment. A uniform extraction algorithm does not make local diagnosis, procedure, or concept coding uniform. Transfer windows do not guarantee observation of outpatient care, events at nonparticipating organizations, or deaths outside recorded encounters. Variable follow-up and potentially informative loss to observation may bias absolute risks despite censoring adjustment and competing-risk methods. Missing death ascertainment can also distort competing-risk estimates. We did not establish covariate balance between included and coverage-excluded patients or quantify the effect of this selection on model performance. The exclusion proportion alone cannot establish comparability or absence of selection bias. Retrospective changes in EHR records and transfer metadata may alter cohort counts, so analyses from different snapshots should not be combined.

The CPT and ICD-9 procedure lists were not exhaustive, and the CPT list included 27 884, a secondary-closure or scar-revision code rather than a new amputation, creating potential for both missed events and misclassification of follow-up procedures (Supplementary Table 15) [20]. ICD-10-PCS has been used for inpatient procedures since 1 October 2015 [21]; the broad 0Y6 detachment rule provides additional ascertainment when those records are available, so omitted-code concerns may be particularly relevant to CPT-only records, including outpatient procedures, and pretransition histories, without guaranteeing complete inpatient capture.

Detailed wound morphology, perfusion, microbiology, treatment, revascularization success, offloading, and adherence were not fully represented. Smoking and prior ulcer history were not included as predictors; their omission does not demonstrate that they lack predictive value. Recorded binary absence need not indicate clinical absence. The older and more comorbid temporal cohort may reflect changing case mix, contributors, or ascertainment; the study cannot distinguish these explanations. Temporal validation is not independent external validation. Aggregate performance does not ensure comparable performance across subgroups: subgroup-to-overall contrasts are descriptive rather than formal difference tests, smaller-group estimates are imprecise, and these analyses do not establish equal clinical benefit. Coefficients are prognostic, not causal. In conclusion, Models C and D offer similar retrospective risk stratification; independent validation, local recalibration, and prospective impact evaluation remain necessary before clinical use.

## Supplementary Material

ofag673_Supplementary_Data

## Supplementary Data

Supplementary materials are available at *Open Forum Infectious Diseases* online. Consisting of data provided by the authors to benefit the reader, the posted materials are not copyedited and are the sole responsibility of the authors, so questions or comments should be addressed to the corresponding author.

## References

[1] Armstrong DG, Boulton AJM, Bus SA. Diabetic foot ulcers and their recurrence. N Engl J Med 2017; 376:2367–75. 10.1056/NEJMra1615439 28614678

[2] Jeffcoate W, Boyko EJ, Game F, Cowled P, Senneville E, Fitridge R. Causes, prevention, and management of diabetes-related foot ulcers. Lancet Diabetes Endocrinol 2024; 12:472–82. 10.1016/S2213-8587(24)00110-4 38824929

[3] Schaper NC, van Netten JJ, Apelqvist J, et al Practical guidelines on the prevention and management of diabetes-related foot disease (IWGDF 2023 update). Diabetes Metab Res Rev 2024; 40:e3657. 10.1002/dmrr.3657 37243927

[4] Senneville E, Albalawi Z, van Asten SA, et al IWGDF/IDSA guidelines on the diagnosis and treatment of diabetes-related foot infections (IWGDF/IDSA 2023). Clin Infect Dis 2023:ciad527. doi:10.1093/cid/ciad527. 37779457

[5] Brennan MB, Powell WR, Kaiksow F, et al Association of race, ethnicity, and rurality with major leg amputation or death among medicare beneficiaries hospitalized with diabetic foot ulcers. JAMA Netw Open 2022; 5:e228399. 10.1001/jamanetworkopen.2022.8399 PMC9024392 35446395

[6] Huang J, Yang J, Qi H, Xu M, Xu X, Zhu Y. Prediction models for amputation after diabetic foot: systematic review and critical appraisal. Diabetol Metab Syndr 2024; 16:126. 10.1186/s13098-024-01360-6 PMC11163763 38858732

[7] Tarabichi Y, Frees A, Honeywell S, et al The cosmos collaborative: a vendor-facilitated electronic health record data aggregation platform. ACI Open 2021; 5:e36–46. 10.1055/s-0041-1731004 PMC8775787 35071993

[8] Centers for Disease Control and Prevention/Agency for Toxic Substances and Disease Registry, Geospatial Research, Analysis, and Services Program . CDC/ATSDR Social Vulnerability Index 2020 Database, United States. Available at: https://www.atsdr.cdc.gov/place-health/php/svi/svi-data-documentation-download.html. Accessed 29 July 2026

[9] US Department of Agriculture, Economic Research Service . Rural-Urban Commuting Area Codes. Updated December 9, 2025. Available at: https://www.ers.usda.gov/data-products/rural-urban-commuting-area-codes/. Accessed 29 July 2026

[10] Putter H, Fiocco M, Geskus RB. Tutorial in biostatistics: competing risks and multi-state models. Stat Med 2007; 26:2389–430. 10.1002/sim.2712 17031868

[11] Blanche P, Dartigues JF, Jacqmin-Gadda H. Estimating and comparing time-dependent areas under receiver operating characteristic curves for censored event times with competing risks. Stat Med 2013; 32:5381–97. 10.1002/sim.5958 24027076

[12] Gerds TA, Schumacher M. Consistent estimation of the expected Brier score in general survival models with right-censored event times. Biom J 2006; 48:1029–40. 10.1002/bimj.200610301 17240660

[13] Vickers AJ, Elkin EB. Decision curve analysis: a novel method for evaluating prediction models. Med Decis Making 2006; 26:565–74. 10.1177/0272989X06295361 PMC2577036 17099194

[14] Collins GS, Moons KGM, Dhiman P, et al TRIPOD + AI statement: updated guidance for reporting clinical prediction models that use regression or machine learning methods. BMJ 2024; 385:e078378. 10.1136/bmj-2023-078378 PMC11019967 38626948

[15] Moons KGM, Damen JAA, Kaul T, et al PROBAST + AI: an updated quality, risk of bias, and applicability assessment tool for prediction models using regression or artificial intelligence methods. BMJ 2025; 388:e082505. 10.1136/bmj-2024-082505 PMC11931409 40127903

[16] Monteiro-Soares M, Hamilton EJ, Russell DA, et al Guidelines on the classification of foot ulcers in people with diabetes (IWGDF 2023 update). Diabetes Metab Res Rev 2024; 40:e3648. 10.1002/dmrr.3648 37179483

[17] Pickwell K, Siersma V, Kars M, et al Predictors of lower-extremity amputation in patients with an infected diabetic foot ulcer. Diabetes Care 2015; 38:852–7. 10.2337/dc14-1598 25665817

[18] Monteiro-Soares M, Dinis-Ribeiro M. A new diabetic foot risk assessment tool: DIAFORA. Diabetes Metab Res Rev 2016; 32:429–35. 10.1002/dmrr.2785 27094098

[19] Røikjer J, Monteiro-Soares M, Walton D, et al External validation of the DIAFORA system to predict lower-extremity amputations in a prospective Danish cohort. Diabet Med 2025; 42:e15443. 10.1111/dme.15443 PMC11635586 39340796

[20] New York State Department of Health . Physician–Surgery Procedure Codes. eMedNY Medicaid Provider Procedure Code Manual. April 2026:65,69. Available at: https://www.emedny.org/ProviderManuals/Physician/PDFS/Physician%20Procedure%20Codes%20Sect5.pdf. Accessed 23 September 2026

[21] Centers for Medicare & Medicaid Services. ICD-10 and Quality Measures. Available at: https://www.cms.gov/medicare/quality/measures/icd-10-and-quality-measures. Accessed 23 September 2026

