# Supplementary material for: Predicting 365-Day Lower-Limb Amputation at Discharge After Diabetes-Related Foot Ulcer Hospitalization: Development and Temporal Validation
Source: Open Forum Infect Dis. 2026 Oct 9;13(10):ofag673. doi: 10.1093/ofid/ofag673 (PMC13714542; doi:10.1093/ofid/ofag673)
Supplement: Supplementary file 1 [file ofag673_supplementary_data.zip › Supplement.docx]

SUPPLEMENTARY MATERIAL

# Predicting 365-Day Lower-Limb Amputation at Discharge After Diabetes-Related Foot Ulcer Hospitalization: Development and Temporal Validation

Manuscript OFID-D-26-01167 | Revised September 23, 2026

Contents: editable Supplementary Tables S1–S17; figure legends, alt text, and Figures S1–S7; and documentation for accompanying machine-readable files SD1–SD11. Detailed model outputs are provided in the accompanying CSV files. Final-refit settings are distinguished from development-fitted settings.

## Model-version distinction

Model performance is reported for the original analysis cohort. The extraction was completed on July 28, 2026, and the final R analysis on July 29, 2026. Model C is the fuller reporting model and Model D the author-defined parsimonious comparator; this terminology does not recommend either for clinical use.

| **File** | **Content** |
| --- | --- |
| SD1_Primary_ModelC_cause_specific_coefficients.csv | Complete final-refit Model C cause-specific coefficients; not the development-fitted coefficient set used for validation. |
| SD2_Design_column_map.csv | Complete design-column specifications for Models C and D. |
| SD3_Final_cumulative_baseline_hazards.csv | Final-refit cumulative baseline hazards. |
| SD4_Source_grouped_CV_performance.csv | Source-grouped out-of-fold performance. |
| SD5_Source_grouped_CV_calibration.csv | Source-grouped out-of-fold calibration. |
| SD6_Temporal_validation_subgroup_performance.csv | All supplied temporal-validation subgroup output, including reportability fields. |
| SD7_Outcome_code_rules.csv | Operational procedure-code inclusion, classification, and anesthesia-exclusion rules from the original SQL. |
| SD8_Continuous_predictor_missingness.csv | Raw and cleaned continuous-predictor counts for the full extract, development, and temporal-validation cohorts. |
| SD9_Final_refit_preprocessing_parameters.csv | 226 final-refit parameter rows: imputation medians, spline knots, categorical levels, and binary coding for final-refit Models C and D and both endpoints. |
| SD10_Categorical_and_binary_distributions.csv | Development/validation category counts, percentages, and the count-source or derivation basis; coded completeness is distinct from clinical ascertainment. |
| SD11_Source_validation_group_counts.csv | Patients and distinct source-validation groups separately for the 2015, development, and temporal-validation cohorts. |

SD1 supplies the complete released Model C coefficient set, with design columns in SD2, final cumulative baseline hazards in SD3, and final-refit preprocessing parameters in SD9 and Table S17. Model D preprocessing and baseline hazards are included for documentation, but its separate coefficients are not supplied. Final-refit parameters were estimated after validation on 468,316 patients and are not the development-fitted parameters used for the reported validation estimates. These research specifications are not a validated clinical calculator.

# Supplementary Table S1. Variable definitions and author-defined predictor sets

| **Predictor / display label** | **Definition** | **Type** | **Models** |
| --- | --- | --- | --- |
| age Age | Age in completed years at index discharge, calculated from birth date; age ≥18 required in SQL; R plausibility range 18–110. | Continuous | A, B, C, D, E |
| creatinine Creatinine | Worst qualifying serum creatinine during the index admission, mg/dL | Continuous | A, B, C, D, E |
| hba1c HbA1c | Worst/available HbA1c during the index admission, percent | Continuous | A, B, C, D, E |
| wbc White blood cell count | Worst qualifying white blood cell count during the index admission, K/uL | Continuous | A, B, C, D, E |
| neutrophil_pct Neutrophil percentage | Worst qualifying neutrophil percentage during the index admission | Continuous | A, B, C, D, E |
| bmi Body mass index | Last qualifying BMI during the index stay, then first or nearest-to-admission value as fallback; extraction accepts BMI 10–100 kg/m². | Continuous | A, B, C, D, E |
| los Length of stay | Index-admission length of stay in days | Continuous | A, B, C, D, E |
| svi Social Vulnerability Index | Zip-level overall CDC/ATSDR SVI percentile rank: 2020 value, with 2018 used when the 2020 value is absent. | Continuous | B, C, E |
| sex_group Sex | Valid sex assigned at birth; legal sex fallback; otherwise other or unknown | Categorical | A, B, C, D, E |
| language_group Preferred language | English, non-English, or unknown preferred language | Categorical | B, C, E |
| insurance_group Insurance | Six-level payer group retaining self-pay/uninsured separately | Categorical | B, C, E |
| ruca_group Rural-urban category | Metropolitan, micropolitan, small town, rural, or unknown RUCA category | Categorical | B, C, E |
| region_group US region | US Census region derived from state | Categorical | B, C, E |
| disposition_group Discharge disposition | Home (including home with home health), skilled nursing facility, rehabilitation, hospice, acute-care transfer, other, or unknown after analysis recoding. | Categorical | C, D, E |
| osteomyelitis Osteomyelitis | Osteomyelitis coded during the index admission | Binary | A, B, C, D, E |
| cellulitis_ssti Cellulitis/soft-tissue infection | Cellulitis or soft-tissue infection coded during the index admission | Binary | A, B, C, D, E |
| necrotizing_fasciitis Necrotizing fasciitis | Necrotizing fasciitis coded during the index admission | Binary | A, B, C, D, E |
| sepsis Sepsis | Sepsis coded during the index admission | Binary | A, B, C, D, E |
| gangrene Gangrene | Gangrene coded during the index admission | Binary | A, B, C, D, E |
| pad Peripheral artery disease | Peripheral artery disease present before or during the index admission | Binary | A, B, C, D, E |
| neuropathy Neuropathy | Peripheral or diabetic neuropathy present before or during the index admission | Binary | A, B, C, D, E |
| esrd_dialysis End-stage kidney disease/dialysis | End-stage kidney disease or dialysis indicator | Binary | A, B, C, D, E |
| prior_amputation Prior amputation | Amputation procedure or acquired-amputation-status code during the 365 days before admission. | Binary | A, B, C, D, E |
| prior_revascularization Prior revascularization | Therapeutic lower-extremity revascularization during the 365-day lookback | Binary | A, B, C, D, E |
| pcp Current general primary care clinician | Current general primary care clinician documented at discharge | Binary | B, C, E |
| deep_debridement Deep debridement | Deep debridement during the index admission | Binary | C, D, E |
| index_revascularization Index-admission revascularization | Therapeutic lower-extremity revascularization during the index admission | Binary | C, D, E |
| home_health_referral Home-health referral | Home-health referral known by discharge | Binary | C, D, E |
| hospice Hospice | Hospice disposition or referral known by discharge | Binary | C, D, E |
| race_group Race | Race group retained for fairness and sensitivity analyses | Categorical | E |
| ethnicity_group Ethnicity | Ethnicity group retained for fairness and sensitivity analyses | Categorical | E |

a Model A: clinical; Model B: clinical + contextual; Model C: clinical + contextual + discharge; Model D: clinical + discharge (parsimonious); Model E: race/ethnicity sensitivity model.

b Continuous predictors use development-cohort median imputation, missingness indicators, and natural cubic splines. Categorical predictors retain specified levels and an explicit unknown category when applicable.

Abbreviations: CDC/ATSDR, Centers for Disease Control and Prevention/Agency for Toxic Substances and Disease Registry; HbA1c, hemoglobin A1c; RUCA, Rural-Urban Commuting Area; SVI, Social Vulnerability Index.

# Supplementary Table S2. Preprocessing rules and modeling settings

| **Parameter** | **Locked value** |
| --- | --- |
| Prediction time | Hospital discharge |
| Prediction horizon | Days 1-365 after discharge |
| Primary outcome | Any subsequent lower-limb amputation |
| Secondary outcome | Major subsequent lower-limb amputation |
| Competing event | Death before the outcome |
| Development cohort | 2016-01-01 through 2024-12-31 |
| Temporal-validation cohort | January 1–June 30, 2025 |
| Historical sensitivity cohort | 2015-01-01 through 2015-12-31 |
| Full reporting model | Model C: clinical + contextual + discharge |
| Parsimonious research candidate | Model D: clinical + discharge |
| Continuous predictors | Development-cohort median imputation, missing indicator, and natural cubic spline |
| Categorical predictors | Specified levels with explicit unknown category; first listed level is the reference |
| Binary predictors | 0/1 coding; 0 is the reference |
| Internal validation | Five-fold source-grouped cross-validation for Models C and D and both outcomes |
| Temporal-validation uncertainty | 500 source-cluster bootstrap replicates with development-fitted models held fixed |
| Subgroup uncertainty | 500 source-cluster bootstrap replicates |
| Infection-focused uncertainty | 500 source-cluster bootstrap replicates |
| Sensitivity-analysis uncertainty | 500 source-cluster bootstrap replicates |
| IPCW weight cap | 99.5th percentile |
| SVI subgroup bands | Fixed national percentile bands: 0-.24, .25-.49, .50-.74, and .75-1.00 |
| Subgroup reportability | n >= 200, >=20 events, >=20 nonevents, and >=5 source-validation groups |
| Race and ethnicity | Excluded from primary model inputs; retained for fairness and sensitivity analyses |
| Quasi-separation handling | Warnings logged; unstable fold-specific terms dropped and models refitted |
| Final model refit | Models C and D refitted after validation in the combined 2016–2024 and January–June 2025 primary cohorts |
| Figure output | Multipart PNG and TIFF, 600 dpi in the analysis script |

a General preprocessing rules are shown here. Table S17 and SD9 supply numerical final-refit settings estimated after validation (n = 468,316), not the development-fitted recipe used for temporal validation. SD2 lists design columns. IPCW, inverse probability of censoring weighting; SVI, Social Vulnerability Index.

# Supplementary Table S3. Cohort characteristics by development and temporal-validation split

| **Characteristic** | **Development 2016–2024 (n = 431,493)** | **Temporal validation January–June 2025 (n = 36,823)** |
| --- | --- | --- |
| Patients | 431,493 | 36,823 |
| Age, years | 65.0 (56.0–75.0) | 68.0 (58.0–77.0) |
| Creatinine, mg/dL | 1.30 (0.91–2.16) | 1.25 (0.90–2.00) |
| HbA1c, % | 7.9 (6.5–10.0) | 7.6 (6.4–9.7) |
| White blood cell count, K/µL | 10.1 (7.7–13.6) | 10.1 (7.7–13.6) |
| Neutrophil percentage | 75.0 (66.5–83.0) | 75.0 (66.3–83.0) |
| Body mass index, kg/m² | 30.4 (25.6–36.4) | 29.4 (25.0–35.1) |
| Length of stay, days | 5.0 (3.0–9.0) | 6.0 (3.0–9.0) |
| Social Vulnerability Index | 0.71 (0.43–0.89) | 0.71 (0.44–0.89) |
| Sex: Female | 148,785 (34.5) | 12,328 (33.5) |
| Sex: Male | 282,689 (65.5) | 24,492 (66.5) |
| Sex: Other or unknown | 19 (0.0) | 3 (0.0) |
| Preferred language: English | 379,479 (87.9) | 30,429 (82.6) |
| Preferred language: Non-English | 52,011 (12.1) | 6,394 (17.4) |
| Preferred language: Unknown | 3 (0.0) | 0 (0.0) |
| Race: White | 260,674 (60.4) | 19,989 (54.3) |
| Race: Black | 69,097 (16.0) | 5,729 (15.6) |
| Race: Asian | 3,905 (0.9) | 406 (1.1) |
| Race: American Indian / Alaska Native | 3,702 (0.9) | 221 (0.6) |
| Race: Multiracial | 48,571 (11.3) | 4,599 (12.5) |
| Race: Other | 15,461 (3.6) | 1,432 (3.9) |
| Race: Unknown | 30,083 (7.0) | 4,447 (12.1) |
| Ethnicity: Not Hispanic or Latino | 351,852 (81.5) | 28,072 (76.2) |
| Ethnicity: Hispanic or Latino | 38,335 (8.9) | 3,449 (9.4) |
| Ethnicity: Unknown | 41,306 (9.6) | 5,302 (14.4) |

**Supplementary Table S3 (continued). Contextual and discharge characteristics**

| **Characteristic** | **Development 2016–2024 (n = 431,493)** | **Temporal validation January–June 2025 (n = 36,823)** |
| --- | --- | --- |
| Insurance: Commercial | 65,481 (15.2) | 6,163 (16.7) |
| Insurance: Medicaid | 61,351 (14.2) | 4,834 (13.1) |
| Insurance: Medicare | 247,285 (57.3) | 21,587 (58.6) |
| Insurance: Other government | 6,451 (1.5) | 828 (2.2) |
| Insurance: Self-pay / uninsured | 8,196 (1.9) | 673 (1.8) |
| Insurance: Other / unknown | 42,729 (9.9) | 2,738 (7.4) |
| RUCA category: Metropolitan | 348,331 (80.7) | 30,138 (81.8) |
| RUCA category: Micropolitan | 41,296 (9.6) | 3,370 (9.2) |
| RUCA category: Small town | 20,803 (4.8) | 1,681 (4.6) |
| RUCA category: Rural | 13,377 (3.1) | 1,071 (2.9) |
| RUCA category: Unknown | 7,686 (1.8) | 563 (1.5) |
| US region: Midwest | 115,790 (26.8) | 8,952 (24.3) |
| US region: Northeast | 84,506 (19.6) | 7,197 (19.5) |
| US region: South | 173,692 (40.3) | 16,261 (44.2) |
| US region: West | 52,471 (12.2) | 4,048 (11.0) |
| US region: Unknown | 5,034 (1.2) | 365 (1.0) |
| SVI band: Q1 | 50,653 (11.7) | 4,256 (11.6) |
| SVI band: Q2 | 77,739 (18.0) | 6,398 (17.4) |
| SVI band: Q3 | 106,538 (24.7) | 9,156 (24.9) |
| SVI band: Q4 | 188,646 (43.7) | 16,485 (44.8) |
| SVI band: Missing | 7,917 (1.8) | 528 (1.4) |
| Discharge disposition: Home (including home health) | 275,832 (63.9) | 23,187 (63.0) |
| Discharge disposition: Skilled nursing facility | 83,940 (19.5) | 7,241 (19.7) |
| Discharge disposition: Rehabilitation | 12,339 (2.9) | 1,082 (2.9) |
| Discharge disposition: Hospice | 9,832 (2.3) | 912 (2.5) |
| Discharge disposition: Acute-care transferᵃ | 0 (0.0) | 0 (0.0) |
| Discharge disposition: Other | 38,232 (8.9) | 3,315 (9.0) |
| Discharge disposition: Unknown | 11,318 (2.6) | 1,086 (2.9) |

a Acute-care transfer is structurally empty in the analytic input: the SQL discharge grouping maps otherwise unclassified dispositions, including acute-care transfers, to Other before R preprocessing. The acute-care-transfer factor level is retained for encoding. Its zero count does not establish that no patients were transferred.

**Supplementary Table S3 (continued). Recorded clinical and care-process indicators**

| **Characteristic** | **Development 2016–2024 (n = 431,493)** | **Temporal validation January–June 2025 (n = 36,823)** |
| --- | --- | --- |
| Current general primary care clinician | 397,992 (92.2) | 34,642 (94.1) |
| Osteomyelitis | 124,666 (28.9) | 11,545 (31.4) |
| Cellulitis / soft-tissue infection | 226,826 (52.6) | 20,231 (54.9) |
| Necrotizing fasciitis | 4,163 (1.0) | 394 (1.1) |
| Sepsis | 98,812 (22.9) | 8,933 (24.3) |
| Gangrene | 67,585 (15.7) | 6,543 (17.8) |
| Peripheral artery disease | 173,619 (40.2) | 15,893 (43.2) |
| Neuropathy | 225,516 (52.3) | 18,797 (51.0) |
| End-stage kidney disease / dialysis | 49,175 (11.4) | 3,775 (10.3) |
| Prior amputation | 67,029 (15.5) | 5,135 (13.9) |
| Prior revascularization | 28,335 (6.6) | 2,362 (6.4) |
| Index deep debridement | 25,496 (5.9) | 2,778 (7.5) |
| Index revascularization | 32,285 (7.5) | 3,342 (9.1) |
| Home-health referral | 18,083 (4.2) | 1,547 (4.2) |
| Hospice disposition / referral | 10,604 (2.5) | 1,004 (2.7) |

Values are median (interquartile range) or n (%), using the corresponding cohort denominator. Continuous missingness is reported in Table S16. Negative binary counts are complements of recorded positive counts; binary indicators do not establish independently adjudicated clinical prevalence. Complete categorical and binary distributions are provided in SD10. RUCA, Rural-Urban Commuting Area; SVI, Social Vulnerability Index.

# Supplementary Table S4. Plausibility cleaning and missing-data rules

| **Variable** | **Lower valid value** | **Upper valid value** | **Action** |
| --- | --- | --- | --- |
| Age, years | 18 | 110 | Outside range converted to missing |
| Creatinine, mg/dL | 0.05 | 100 | Outside range converted to missing |
| HbA1c, % | 3 | 30 | Outside range converted to missing |
| White blood cell count, K/uL | 0.1 | 1000 | Outside range converted to missing |
| Neutrophil percentage | 0 | 100 | Outside range converted to missing |
| Body mass index, kg/m2 | 10 | 150 | Outside range converted to missing |
| Length of stay, days | 0 | 730 | Outside range converted to missing |
| Social Vulnerability Index | 0 | 1 | Outside range converted to missing |

a For continuous predictors, values outside the stated range were converted to missing before model preprocessing.

b Missing continuous values were imputed with development-cohort medians and accompanied by missingness indicators. Categorical missingness was retained as an explicit unknown level.

c The SQL calculates age at discharge and requires age ≥18 for eligibility. The original extract contained no missing age before cleaning; five ages above 110 were converted to missing in R (four development; one temporal validation). The age_missing column follows the same preprocessing convention as the other continuous missingness indicators; it is not an enrollment rule.

d BMI values were restricted to 10–100 kg/m² during SQL extraction. R subsequently applied the broader 10–150 kg/m² plausibility range shown here; this second check did not restore values rejected during extraction.

Continuous missingness counts and the distinction between source absence and plausibility cleaning are reported in Table S16.

# Supplementary Table S5. Data dictionary for the final-refit coefficient file (SD1)

| **Field** | **Description** |
| --- | --- |
| design_column | Final model-matrix column, including spline bases, missing indicators, categorical dummy variables, and binary terms |
| model | Specified model identifier |
| cause | Cause-specific endpoint: amputation, major amputation, or death before the corresponding amputation endpoint |
| coefficient / standard_error | Log hazard coefficient and standard error |
| hazard_ratio / ci_lower / ci_upper | Exponentiated coefficient and 95% confidence interval |
| p_value | Wald test p value |
| source_variable / term_type | Original predictor and encoded term class |
| estimable / coefficient_status | Indicator of an estimated coefficient and explicit handling of dropped/nonestimable terms; fixed-zero terms contribute 0 to xβ |

Notes: SD1 includes all 70 Model C design columns per cause-specific fit; nonestimable columns are fixed at coefficient 0.0 and explicitly flagged in the CSV.

# Supplementary Table S6. Data dictionary for the design-column map (SD2)

| **Field** | **Description** |
| --- | --- |
| model | Specified model identifier |
| design_column | Exact model-matrix column name used during fitting and prediction |
| source_variable | Original clinical, contextual, or discharge predictor |
| term_type | Continuous spline, missing indicator, categorical dummy, or binary term |
| reference level / coding metadata | Reference category and encoding information needed to reproduce the design matrix |

Notes: The design map defines matrix construction and column order. No mean-centering is applied to the linear predictor.

SD3 supplies cumulative baseline hazards for final-refit Models C and D at 0.5 day and days 1–365. These are paired with uncentered cause-specific linear predictors exp(xβ). Do not subtract a cohort-average linear predictor. SD9 and Table S17 provide the corresponding final-refit preprocessing parameters; SD2 supplies design-column names and order.

# Supplementary Table S7. Data dictionary for source-grouped cross-validation performance (SD4)

| **Field** | **Description** |
| --- | --- |
| model / outcome | Models C and D evaluated for any and major amputation |
| dataset | Development source-grouped out-of-fold predictions from 5 folds |
| n / events / competing_deaths | Out-of-fold sample and event counts |
| observed_cif / mean_predicted_risk / O/E ratio | Calibration-in-the-large quantities at 365 days |
| AUC / AUPRC / Brier score | IPCW discrimination and overall accuracy metrics |
| calibration_intercept / calibration_slope | Out-of-fold calibration estimates |

Notes: SD4 contains complete out-of-fold Model C and Model D performance estimates; development-fitted models were not refitted to validation outcomes.

# Supplementary Table S8. Data dictionary for source-grouped cross-validation calibration (SD5)

| **Field** | **Description** |
| --- | --- |
| model / outcome | Models C and D evaluated for any and major amputation |
| risk_group | Decile of out-of-fold predicted 365-day risk |
| n / mean_predicted / median_predicted | Decile size and predicted-risk summaries |
| observed_cif / raw_event_n | Aalen-Johansen observed cumulative incidence and raw event count |

Notes: Observed CIF values are Aalen-Johansen estimates at 365 days; risk groups are out-of-fold predicted-risk deciles.

# Supplementary Table S9. Paired temporal-validation comparisons of Models C and D

| **Outcome** | **Metric** | **Model C minus Model D** | **95% CI** |
| --- | --- | --- | --- |
| Any amputation | AUC | -0.00140 | -0.00243 to -0.00028 |
| Any amputation | AUPRC | -0.00021 | -0.00154 to 0.00125 |
| Any amputation | Brier score | 0.00002 | -0.00013 to 0.00017 |
| Major amputation | AUC | 0.00098 | -0.00138 to 0.00420 |
| Major amputation | AUPRC | -0.00135 | -0.00506 to 0.00184 |
| Major amputation | Brier score | 0.00001 | -0.00009 to 0.00014 |

a Positive values favor Model C for AUC and AUPRC; negative values favor Model C for Brier score because lower is better.

b Confidence intervals are from 500 paired source-cluster bootstrap replicates using the same resampled validation sources for both models. Abbreviations: AUC, area under the receiver operating characteristic curve; AUPRC, area under the precision-recall curve; CI, confidence interval.

# Supplementary Table S10. Model C risk-stratum operating characteristics

| **Outcome** | **Selected risk fraction** | **Sensitivity** | **Positive predictive value** |
| --- | --- | --- | --- |
| Any amputation | Top 10% | 0.243 | 0.449 |
| Any amputation | Top 20% | 0.406 | 0.378 |
| Major amputation | Top 10% | 0.314 | 0.195 |
| Major amputation | Top 20% | 0.496 | 0.155 |

a The selected fraction is the proportion of the temporal-validation cohort with the highest predicted risk under Model C.

b Positive predictive value is the Aalen-Johansen 365-day cumulative incidence within the selected risk stratum; it is not the crude event count divided by the number selected.

# Supplementary Table S11. Subgroup reportability and analysis dimensions

| **Subgroup dimension** | **Levels represented** | **Outcomes** | **Uncertainty/reporting rule** |
| --- | --- | --- | --- |
| Sex | Female; male; other/unknown | Any and major amputation | Report estimates only when specified sample/event thresholds are met |
| Race | White; Black; Asian; American Indian/Alaska Native; multiracial; other; unknown | Any and major amputation | 500 source-cluster bootstrap replicates for reportable cells |
| Ethnicity | Hispanic/Latino; not Hispanic/Latino; unknown | Any and major amputation | 500 source-cluster bootstrap replicates |
| Preferred language | English; non-English | Any and major amputation | 500 source-cluster bootstrap replicates |
| Insurance | Commercial; Medicaid; Medicare; other government; other/unknown; self-pay/uninsured | Any and major amputation | 500 source-cluster bootstrap replicates |
| RUCA | Metropolitan; micropolitan; small town; rural; unknown | Any and major amputation | 500 source-cluster bootstrap replicates |
| SVI band | Q1, Q2, Q3, Q4, missing | Any and major amputation | Fixed national percentile cut points; 500 bootstrap replicates when reportable |
| US region | Midwest; Northeast; South; West; unknown | Any and major amputation | 500 source-cluster bootstrap replicates when reportable |

a Nonreportable cells remain visible in the complete locked output table with reportability flags but are omitted from inferential forest plots.

Data SD6 includes every subgroup for both endpoints, with sample sizes, event counts, discrimination, calibration, uncertainty, and reportability flags. Forest plots include reportable estimates only. Subgroup interpretation uses overall Model C AUCs of 0.710 and 0.737 and O/E ratios of 1.07 and 0.95 for any and major amputation, respectively; these descriptive comparisons are not formal subgroup-versus-overall tests.

# Supplementary Table S12. Infection-focused temporal-validation analyses

| **Infection subgroup** | **Outcome** | **Model** | **n** | **AUC (95% CI)** | **AUPRC (95% CI)** | **O/E ratio (95% CI)** |
| --- | --- | --- | --- | --- | --- | --- |
| Any coded infection phenotype | Any amputation | C | 27,644 | 0.697 (0.687-0.707) | 0.387 (0.371-0.405) | 1.102 (1.065-1.139) |
| Any coded infection phenotype | Any amputation | D | 27,644 | 0.698 (0.689-0.708) | 0.387 (0.371-0.405) | 1.104 (1.065-1.140) |
| Any coded infection phenotype | Major amputation | C | 27,644 | 0.734 (0.720-0.746) | 0.181 (0.168-0.199) | 0.961 (0.909-1.013) |
| Any coded infection phenotype | Major amputation | D | 27,644 | 0.731 (0.715-0.744) | 0.180 (0.165-0.200) | 0.968 (0.908-1.019) |
| Osteomyelitis | Any amputation | C | 11,545 | 0.656 (0.643-0.670) | 0.393 (0.368-0.420) | 1.091 (1.036-1.140) |
| Osteomyelitis | Any amputation | D | 11,545 | 0.656 (0.644-0.670) | 0.392 (0.367-0.419) | 1.092 (1.036-1.139) |
| Osteomyelitis | Major amputation | C | 11,545 | 0.696 (0.676-0.716) | 0.172 (0.153-0.199) | 0.967 (0.894-1.044) |
| Osteomyelitis | Major amputation | D | 11,545 | 0.692 (0.672-0.712) | 0.170 (0.151-0.200) | 0.973 (0.896-1.054) |

a Models C and D were fitted in development and evaluated without refitting in the indicated temporal-validation subgroups.

b Confidence intervals are from 500 source-cluster bootstrap replicates.

Abbreviations: AUC, area under the receiver operating characteristic curve; AUPRC, area under the precision-recall curve; O/E, observed-to-expected.

# Supplementary Table S13. Additional sensitivity analyses

| **Analysis** | **Outcome** | **Model** | **n** | **AUC** | **O/E ratio** | **Brier score** |
| --- | --- | --- | --- | --- | --- | --- |
| Exclude hospice | Any amputation | C | 35,819 | 0.705 | 1.063 | 0.145 |
| Exclude hospice | Any amputation | D | 35,819 | 0.706 | 1.066 | 0.145 |
| Exclude hospice | Major amputation | C | 35,819 | 0.737 | 0.943 | 0.058 |
| Exclude hospice | Major amputation | D | 35,819 | 0.734 | 0.950 | 0.058 |
| Development includes 2015 | Any amputation | C | 36,823 | 0.710 | 1.070 | 0.142 |
| Development includes 2015 | Any amputation | D | 36,823 | 0.711 | 1.073 | 0.142 |
| Development includes 2015 | Major amputation | C | 36,823 | 0.739 | 0.944 | 0.057 |
| Development includes 2015 | Major amputation | D | 36,823 | 0.736 | 0.952 | 0.057 |
| Race/ethnicity added | Any amputation | E | 36,823 | 0.710 | 1.068 | 0.142 |
| Race/ethnicity added | Major amputation | E | 36,823 | 0.741 | 0.947 | 0.057 |

a The table reports the sensitivity-analysis sample sizes, AUCs, O/E ratios, and Brier scores used in the manuscript. The same 500-replicate source-cluster bootstrap framework was used for uncertainty estimation.

b Model E is the author-defined race/ethnicity sensitivity model (Model C plus race and ethnicity). Abbreviations: AUC, area under the receiver operating characteristic curve; O/E, observed-to-expected.

# Supplementary Table S14. Locked-cohort and bootstrap reconciliation

| **Check** | **Expected** | **Observed** | **Difference/failures** | **Status** |
| --- | --- | --- | --- | --- |
| First qualifying discharges minus index exclusions | 506,629 | 506,629 | 0 | Pass |
| Eligible minus source-coverage exclusions | 496,927 | 496,927 | 0 | Pass |
| Final cohort equals analysis-extract rows | 496,927 | 496,927 | 0 | Pass |
| 2015 sensitivity plus primary cohort | 496,927 | 496,927 | 0 | Pass |
| Development plus temporal validation | 468,316 | 468,316 | 0 | Pass |
| Main metric bootstrap replicates | 500; >=450 finite required | All reportable metrics met threshold | 0 failures | Pass |
| Paired comparison bootstrap replicates | 500; >=450 finite required | All comparisons met threshold | 0 failures | Pass |
| Subgroup bootstrap replicates | 500; >=450 finite required | All reportable metrics met threshold | 0 failures | Pass |
| Infection/sensitivity bootstrap replicates | 500; >=450 finite required | All reportable metrics met threshold | 0 failures | Pass |
| Calibration intercept/slope bootstrap replicates | 500; >=450 finite required | All 4 models and both outcomes met threshold | 0 failures | Pass |

a Sex derivation yielded 325,418 male, 171,486 female, and 23 other/unknown patients in the final cohort; 151,261 values came from sex assigned at birth and 345,643 from legal-sex fallback.

b Cox term-stability actions, fold-specific term drops, and deterministic refits were audited during the locked analysis; all reportable performance outputs met the specified bootstrap-completeness threshold.

**Supplementary Table S14 (continued). Cohort source-validation groups**

| **Cohort** | **Patients** | **Distinct source-validation groups** |
| --- | --- | --- |
| Historical sensitivity, 2015 | 28,611 | 130 |
| Development, 2016–2024 | 431,493 | 262 |
| Temporal validation, January–June 2025 | 36,823 | 250 |

Cohort-specific counts are also supplied in SD11. These describe analytic resampling groups, not individual hospitals. Single-source encounters retain a source-specific group; multisource encounters share one grouping category, and absent source linkage is assigned an unknown-source category. Counts must not be summed to obtain a unique total and do not establish the overlap of contributing groups between periods.

# Supplementary Table S15. Implemented outcome-code and ascertainment specification

## A. Procedure-code rules

| **Code system** | **Implemented class** | **Accepted values or rule** |
| --- | --- | --- |
| CPT | Minor | 28800, 28805, 28810, 28820, 28825 |
| CPT | Major | 27590, 27591, 27592, 27880, 27882, 27884 |
| ICD-9-CM procedure | Minor | 84.10, 84.11, 84.12 (normalized: 8410, 8411, 8412) |
| ICD-9-CM procedure | Major | 84.13, 84.14, 84.15, 84.16, 84.17 (normalized: 8413, 8414, 8415, 8416, 8417) |
| ICD-10-PCS | Any | Any normalized value beginning with 0Y6. |
| ICD-10-PCS | Major | 0Y6 prefix and fourth character in {2, 3, 4, 7, 8, C, D, F, G, H, J}. |
| ICD-10-PCS | Minor | 0Y6 prefix and fourth character in {M, N, P, Q, R, S, T, U, V, W, X, Y}. |
| All code systems | Anesthesia exclusion | Exclude when any of the five code fields is a five-character string with integer value 100–1999, or combined procedure text contains ANESTH, begins with ANES plus a space, or contains \|ANES plus a space. |

a Any-amputation CPT and ICD-9-CM matches are the union of their minor and major lists. The major classification is evaluated first. A 0Y6 match outside the listed fourth-character groups remains an any-amputation match with unclassified level.

b Codes are trimmed, uppercased, and stripped of periods and spaces. CPT values are matched across the available procedure-event and procedure-dictionary code fields; ICD procedure values are matched in the event code and primary or additional dictionary codes. The ICD-10-PCS definition is a prefix/body-part rule, not an enumerated seven-character whitelist.

c Candidate procedures were retrieved before exact-code reclassification removed description-only matches and applied the anesthesia override; patient-level first-event dates were then assembled. The table reproduces the implemented rules, including CPT 27884, rather than a retrospectively expanded code set.

d The CPT list does not include 27290, 27295, 27594, 27596, 27598, 27881, 27886, 27888, or 27889; ICD-9-CM procedure codes 84.18 and 84.19 are also absent. These omitted codes encompass different amputation, re-amputation, and closure/revision procedures and are not interchangeable indicators of new limb loss. The included CPT 27884 describes secondary closure or scar revision, not a new amputation; treating it as a qualifying event can therefore misclassify follow-up care. These code-set limitations may affect index exclusions, prior-procedure predictors, and subsequent outcomes.

Abbreviations: CPT, Current Procedural Terminology; ICD, International Classification of Diseases; PCS, Procedure Coding System.

Coding context: ICD-10-PCS inpatient coding began October 1, 2015. The 0Y6 rule broadens ascertainment when corresponding records are present, but does not guarantee complete capture; omitted codes may particularly affect CPT-only outpatient records and pretransition history. Their effect on reported estimates was not quantified (main-manuscript references 20 and 21).

# Supplementary Table S15 (continued). Timing, eligibility, and source coverage

| **Component** | **Implementation** |
| --- | --- |
| Index and subsequent procedures | Index: procedure time from admission through discharge. Subsequent: strictly after discharge and within 365 days. First any and first major events are selected separately. |
| Procedure record eligibility | Records are not deleted, inferred, or flagged as unsuccessful attempts. Start time is used, with end time as fallback. |
| Observed endpoint | Event must occur within available follow-up and on or before recorded death. Death strictly before the endpoint is competing; same-calendar-date ties favor amputation. |
| Observation end | Earliest of day 365, June 30, 2026, source-complete transfer end, and last known observation including a later recorded death. With no later observation, discharge is the fallback. |
| Multisource interval | Use the latest complete-transfer start and earliest complete-transfer end across linked sources. A missing boundary for any source makes that conservative boundary missing. |
| Source eligibility | Both boundaries must be present. Index admission date must be on/after the start and discharge date on/before the end. The entire stay must lie inside the interval. |
| Source grouping | A single-source encounter is grouped by its source key. Multisource encounters are grouped as MULTISOURCE. Source grouping is used for resampling/validation, not as a predictor. |
| Prior observability | A prior-year encounter was not required. Prior-year encounter and source-lookback flags were retained for observability assessment without imposing a primary eligibility restriction. |
| Extraction and analysis periods | SQL extraction includes 2015–2024 development-labelled discharges; the primary R analysis uses 2016–2024, reserving 2015 for historical sensitivity analysis. |

Extraction and analysis periods are distinguished: the extraction includes 2015, whereas the primary analysis reserves that year for historical sensitivity analysis. The archived SQL specifies the timing, eligibility, source grouping, and follow-up rules summarized here.

# Supplementary Table S16. Predictor missingness and encoded completeness before imputation

## A. Missing observations after plausibility cleaning, n (%)

| **Predictor** | **Full extract (n = 496,927)** | **Development (n = 431,493)** | **Temporal validation (n = 36,823)** |
| --- | --- | --- | --- |
| Age, years | 5 (0.0010) | 4 (0.0009) | 1 (0.0027) |
| Creatinine, mg/dL | 65,418 (13.16) | 57,422 (13.31) | 3,069 (8.33) |
| HbA1c, % | 274,384 (55.22) | 238,311 (55.23) | 20,044 (54.43) |
| White blood cell count, K/µL | 182,583 (36.74) | 156,952 (36.37) | 12,117 (32.91) |
| Neutrophil percentage | 261,019 (52.53) | 224,353 (51.99) | 18,276 (49.63) |
| Body mass index, kg/m² | 142,239 (28.62) | 124,371 (28.82) | 10,800 (29.33) |
| Length of stay, days | 20 (0.0040) | 19 (0.0044) | 0 (0.00) |
| Social Vulnerability Index | 9,312 (1.87) | 7,917 (1.83) | 528 (1.43) |

a Full extract includes the 28,611 discharges in 2015; development includes 2016–2024 and temporal validation January–June 2025. Percentages use the column denominators. Values are missing before median imputation, not after it.

b Counts describe missing model-input values before imputation, using the stated cohort denominators and plausibility limits; they are not post-imputation counts.

c Panel C distinguishes explicit unknown levels from combined other/unknown categories. Panel D reports completeness of derived binary encoding, not complete clinical assessment. A coded zero can represent no qualifying record. HbA1c, hemoglobin A1c; K/µL, thousands per microliter.

# Supplementary Table S16 (continued). Raw-to-cleaned continuous counts

| **Predictor** | **Raw missing, n** | **Changed to missing, n** | **After cleaning, n** |
| --- | --- | --- | --- |
| Age, years | 0 | 5 | 5 |
| Creatinine, mg/dL | 64,957 | 461 | 65,418 |
| HbA1c, % | 273,542 | 842 | 274,384 |
| White blood cell count, K/µL | 182,583 | 0 | 182,583 |
| Neutrophil percentage | 261,019 | 0 | 261,019 |
| Body mass index, kg/m² | 142,239 | 0 | 142,239 |
| Length of stay, days | 0 | 20 | 20 |
| Social Vulnerability Index | 9,312 | 0 | 9,312 |

The full-extract denominator is 496,927. Five age values above 110 were converted to missing; raw age had no missing values and ranged from 18 to 134. Missingness indicators were generated under the shared continuous-preprocessing rule. Retaining a zero coefficient for a nonestimable age_missing term does not imply that no ages were missing.

Fitted final-refit settings are provided separately in Table S17. Descriptive medians in Table S3 are not substituted for fitted imputation parameters.

**Supplementary Table S16 (continued). Categorical unknown and non-specific levels**

| **Predictor** | **Reported level** | **Development n (%)** | **Temporal validation n (%)** | **Interpretation** |
| --- | --- | --- | --- | --- |
| Sex | Other or unknown | 19 (0.00) | 3 (0.01) | Combined level; not pure missingness |
| Preferred language | Unknown | 3 (0.00) | 0 (0.00) | Residual after English/non-English; earlier source audit corroborates 3 |
| Race | Unknown | 30,083 (6.97) | 4,447 (12.08) | Unknown level; Other is not counted as missing |
| Ethnicity | Unknown | 41,306 (9.57) | 5,302 (14.40) | Unknown level |
| Insurance | Other / unknown | 42,729 (9.90) | 2,738 (7.44) | Combined level; cannot isolate pure missingness |
| RUCA category | Unknown | 7,686 (1.78) | 563 (1.53) | Unknown level |
| US region | Unknown | 5,034 (1.17) | 365 (0.99) | Unknown level |
| Discharge disposition | Unknown | 11,318 (2.62) | 1,086 (2.95) | Unknown level |

These are counts in the reported categories, not a claim that raw categorical fields are otherwise clinically complete. Other/unknown sex and insurance cannot be decomposed into separate missing and nonmissing components from these aggregate counts. The three development unknown-language records equal the cohort denominator minus the reported English and non-English counts. Missing continuous SVI is already reported in Panel A. Complete distributions are provided in SD10.

**Supplementary Table S16 (continued). Completeness of the derived binary representation**

| **Derived binary predictor** | **Unencoded observations Development (n = 431,493)** | **Unencoded observations Temporal validation (n = 36,823)** |
| --- | --- | --- |
| Current general primary care clinician | 0 | 0 |
| Osteomyelitis | 0 | 0 |
| Cellulitis / soft-tissue infection | 0 | 0 |
| Necrotizing fasciitis | 0 | 0 |
| Sepsis | 0 | 0 |
| Gangrene | 0 | 0 |
| Peripheral artery disease | 0 | 0 |
| Neuropathy | 0 | 0 |
| End-stage kidney disease / dialysis | 0 | 0 |
| Prior amputation | 0 | 0 |
| Prior revascularization | 0 | 0 |
| Index deep debridement | 0 | 0 |
| Index revascularization | 0 | 0 |
| Home-health referral | 0 | 0 |
| Hospice disposition / referral | 0 | 0 |

For each binary variable, the counts coded 0 and 1 sum to the cohort denominator, leaving no observations unencoded in the derived representation. This does not mean that the condition was assessed in every patient: the extraction codes absence of a qualifying record as zero. Negative counts are complements of the positive counts. Clinical nonassessment cannot be quantified from these aggregate flags.

**Supplementary Table S17. Final-refit preprocessing specification**

**A. Continuous predictors: imputation medians and natural-spline knots**

| **Predictor** | **Imputation median** | **Internal knots (in listed order)** | **Boundary knots** | **Models** |
| --- | --- | --- | --- | --- |
| Age, years | 66 | 60, 72 | 18, 110 | C and D |
| Creatinine, mg/dL | 1.3 | 1.1, 1.57 | 0.1, 100 | C and D |
| HbA1c, % | 7.8 | 7.8, 7.8 | 3.4, 25.6 | C and D |
| White blood cell count, K/µL | 10.1 | 10.1, 10.1 | 0.1, 250 | C and D |
| Neutrophil percentage | 75 | 75, 75 | 0, 100 | C and D |
| Body mass index, kg/m² | 30.28 | 29.68, 30.91 | 10.0781, 99.79 | C and D |
| Length of stay, days | 5 | 4, 7 | 1, 716 | C and D |
| Social Vulnerability Index | 0.7059 | 0.5473, 0.8302 | 0, 1 | C only |

The complete 226-row final-refit preprocessing specification is supplied in SD9. Parameters were fitted after validation in the combined primary cohort of 468,316 patients (2016–2024 and January–June 2025). Within each model, the settings are identical for the any- and major-amputation endpoints. These parameters accompany the final-refit coefficients and baseline hazards; they were not used to calculate the reported temporal-validation results.

Natural cubic splines use 3 degrees of freedom with intercept = FALSE. Repeated internal knot values for HbA1c (7.8, 7.8), white blood cell count (10.1, 10.1), and neutrophil percentage (75, 75) are part of the fitted specification and were retained without deduplication. Boundary knots are fitted values, distinct from the plausibility limits in Table S4. The final-refit age imputation median is 66; the descriptive development-cohort median is 65.

**Supplementary Table S17 (continued). Categorical levels and reference categories**

| **Predictor** | **Levels, in recipe order** | **Reference** | **Models** |
| --- | --- | --- | --- |
| Sex | Female; Male; Other or unknown | Female | C and D |
| Preferred language | English; Non-English; Unknown | English | C |
| Insurance | Commercial; Medicaid; Medicare; Other government; Self-pay / uninsured; Other / unknown | Commercial | C |
| RUCA category | Metropolitan; Micropolitan; Small town; Rural; Unknown | Metropolitan | C |
| US region | Midwest; Northeast; South; West; Unknown | Midwest | C |
| Discharge disposition | Home (including home health); Skilled nursing facility; Rehabilitation; Hospice; Acute-care transfer; Other; Unknown | Home (including home health) | C and D |

The first listed level is the reference category. Machine-readable level names and their order are provided in SD9. Acute-care transfer is a retained but structurally empty level because the SQL discharge grouping does not separately encode transfers; see the explanatory footnote to Table S3.

**Supplementary Table S17 (continued). Binary coding and model pairing**

| **Predictor** | **Coding** | **Models** |
| --- | --- | --- |
| Osteomyelitis | 0/1; reference = 0 | C and D |
| Cellulitis / soft-tissue infection | 0/1; reference = 0 | C and D |
| Necrotizing fasciitis | 0/1; reference = 0 | C and D |
| Sepsis | 0/1; reference = 0 | C and D |
| Gangrene | 0/1; reference = 0 | C and D |
| Peripheral artery disease | 0/1; reference = 0 | C and D |
| Neuropathy | 0/1; reference = 0 | C and D |
| End-stage kidney disease / dialysis | 0/1; reference = 0 | C and D |
| Prior amputation | 0/1; reference = 0 | C and D |
| Prior revascularization | 0/1; reference = 0 | C and D |
| Current general primary care clinician | 0/1; reference = 0 | C only |
| Index deep debridement | 0/1; reference = 0 | C and D |
| Index revascularization | 0/1; reference = 0 | C and D |
| Home-health referral | 0/1; reference = 0 | C and D |
| Hospice disposition / referral | 0/1; reference = 0 | C and D |

A missingness-indicator design column accompanies each continuous predictor, as listed in SD2. Final Model C coefficients and nonestimable-term handling are provided in SD1. Model D recipes are documented here, but its coefficients are not supplied; SD2, SD3, and SD9 alone do not release a complete Model D scoring model. The descriptive category counts in SD10 are not substituted for factor references or fitted recipe values.

# SUPPLEMENTARY FIGURE ALT TEXT

Figure S1. Horizontal bar chart ranking Model C predictors for any amputation. Gangrene is the dominant predictor, followed by peripheral artery disease, length of stay, index-admission revascularization, osteomyelitis, discharge disposition, and cellulitis/soft-tissue infection.

Figure S2. Horizontal bar chart ranking Model C predictors for major amputation. Gangrene and peripheral artery disease dominate, followed by prior revascularization, prior amputation, end-stage kidney disease/dialysis, discharge disposition, and index-admission revascularization.

Figure S3. Two calibration panels compare Model C and Model D using out-of-fold predictions from source-grouped cross-validation. Points plot observed cumulative incidence against mean predicted 365-day risk, with a diagonal reference for perfect calibration.

Figure S4. Forest plot of reportable any-amputation AUCs and bootstrap confidence intervals, with a dotted line at the overall Model C AUC of 0.710. Estimates range from 0.604 for self-pay/uninsured to 0.732 for Medicare. The six lowest estimates are self-pay/uninsured, American Indian/Alaska Native (0.620), unknown race (0.645), unknown ethnicity (0.654), commercial insurance (0.665), and non-English language (0.673). Full subgroup results and reportability flags are in SD6.

Figure S5. Forest plot of reportable major-amputation AUCs and bootstrap confidence intervals, with a dotted line at the overall Model C AUC of 0.737. Estimates range from 0.583 for self-pay/uninsured to 0.801 for rural patients. Other/unknown insurance (0.695), unknown race (0.697), commercial insurance (0.704), and non-English language (0.706) are below the overall point estimate; Asian (0.771) and Medicare (0.762) estimates are above it. Intervals are wide in some smaller groups; SD6 contains all results.

Figure S6. Forest plot of reportable any-amputation observed-to-expected risk ratios with bootstrap confidence intervals. The dotted line marks ideal calibration at 1.0; the overall Model C ratio is 1.07. Other-government insurance (1.35) and other/unknown insurance (1.28) show more underprediction than the overall point estimate. Missing SVI (0.84) and American Indian/Alaska Native (0.88) have lower point estimates. These are descriptive subgroup-to-overall comparisons, not formal difference tests.

Figure S7. Forest plot of reportable major-amputation observed-to-expected risk ratios with bootstrap confidence intervals. The dotted line marks ideal calibration at 1.0; the overall Model C ratio is 0.95. Ratios are 1.12 for Black patients, 0.86 for White patients, and 1.45 for other-government insurance. These show descriptive variation around the overall estimate; some smaller groups have wide intervals. The plot does not establish differences in benefit from model-guided care.

# Supplementary Figure S1

Supplementary Figure S1. Model C variable-importance display for any amputation. Bars show joint Wald chi-square statistics for the displayed predictor groups; larger values indicate stronger statistical evidence of association with the modeled cause-specific hazard. Splines and multilevel variables contribute multiple parameters. These statistics do not give direction, causal effect, or the change in discrimination after removing a variable. Model C contains clinical, contextual, and discharge predictors.

# Supplementary Figure S2

Supplementary Figure S2. Model C variable-importance display for major amputation. Bars show joint Wald chi-square statistics, with splines and multilevel variables contributing multiple parameters. The ranking is not a ranking of causal effects or intervention targets. Model C contains clinical, contextual, and discharge predictors.

# Supplementary Figure S3

Supplementary Figure S3. Source-grouped cross-validation calibration for Models C and D. Panels A and B show observed Aalen-Johansen cumulative incidence against mean out-of-fold predicted risk for any and major amputation, respectively. Model C is the clinical + contextual + discharge model; Model D is the clinical + discharge (parsimonious) model.

# Supplementary Figure S4

Supplementary Figure S4. Temporal-validation subgroup discrimination for any amputation. Points are subgroup IPCW 365-day AUC estimates and horizontal lines are 500-replicate source-cluster bootstrap confidence intervals. The dotted vertical line is the overall Model C AUC. Abbreviations: AUC, area under the receiver operating characteristic curve; IPCW, inverse probability of censoring weighting; RUCA, Rural-Urban Commuting Area; SVI, Social Vulnerability Index.

# Supplementary Figure S5

Supplementary Figure S5. Temporal-validation subgroup discrimination for major amputation. Points are subgroup IPCW 365-day AUC estimates and horizontal lines are 500-replicate source-cluster bootstrap confidence intervals. The dotted vertical line is the overall Model C AUC. Abbreviations: AUC, area under the receiver operating characteristic curve; IPCW, inverse probability of censoring weighting; RUCA, Rural-Urban Commuting Area; SVI, Social Vulnerability Index.

# Supplementary Figure S6

Supplementary Figure S6. Temporal-validation subgroup calibration for any amputation. Points are subgroup observed-to-expected ratios and horizontal lines are 500-replicate source-cluster bootstrap confidence intervals. The dotted vertical line indicates ideal calibration at 1.0; the overall Model C O/E ratio is 1.07 and provides the cohort benchmark for descriptive subgroup comparisons. Abbreviations: O/E, observed-to-expected; RUCA, Rural-Urban Commuting Area; SVI, Social Vulnerability Index.

# Supplementary Figure S7

Supplementary Figure S7. Temporal-validation subgroup calibration for major amputation. Points are subgroup observed-to-expected ratios and horizontal lines are 500-replicate source-cluster bootstrap confidence intervals. The dotted vertical line indicates ideal calibration at 1.0; the overall Model C O/E ratio is 0.95 and provides the cohort benchmark for descriptive subgroup comparisons. Abbreviations: O/E, observed-to-expected; RUCA, Rural-Urban Commuting Area; SVI, Social Vulnerability Index.
